# Spatial and temporal dynamics of cancer-associated fibroblast niches in breast cancer

**DOI:** 10.1186/s13058-025-02183-7

**Published:** 2026-01-11

**Authors:** Jessica Pantaleo, Jonas Sjölund, Paulina Bolivar, Matteo Bocci, Bengt Phung, Maria Malmberg, Göran B. Jönsson, Kristian Pietras

**Affiliations:** 1 Department of Laboratory Medicine, Division of Translational Cancer Research, Lund University Cancer Centre, Lund University, Lund, Sweden; 2 Department of Clinical Sciences, Division of Oncology, Lund University, Lund, Sweden; 3 SciLifeLab, Department of Laboratory Medicine, Lund University, Lund, Sweden

**Keywords:** Breast cancer, Tumor microenvironment, Spatial biology, Cancer-associated fibroblasts

## Abstract

**Background:**

Cancer-associated fibroblasts (CAFs) are the main constituents of the tumor microenvironment. Several studies have delineated CAF heterogeneity in different types of tumors, however, it is still unknown how the distinct CAF transcriptional profiles are established during tumor progression.

**Methods:**

We reanalyzed a previously published single-cell RNA-sequencing dataset of MMTV-PyMT tumors at higher resolution using Seurat , and CytoTRACE to characterize CAF subtypes and their differentiation states. Wilcoxon rank sum test was applied for differential gene expression. Multiplex immunostaining (Akoya PhenoImager HT) was performed on 38 murine mammary tumors from MMTV-PyMT mice to identify the distinct CAF subtypes. Whole-slide imaging and spatial analysis were conducted using QuPath and Cellpose , followed by neighborhood clustering and interaction mapping with CytoMAP . Cellular distances from CAFs to immune, tumor, and endothelial cells were quantified using SPIAT and Wilcoxon tests for comparisons. In parallel, human spatial transcriptomics data from the 10X Genomics Xenium platform were integrated for cross-species validation.

**Results:**

Here, by single cell RNA-sequencing and multiplex immunostaining, we identify six CAF substates. Spatial analysis on immunostained murine mammary tumors and human spatial transcriptomics data outlined temporal changes in stromal composition and the existence of distinct functional niches enriched with different CAF substates. Immunomodulatory CAFs co-localized with immune cells while myofibroblastic CAFs formed a shield around the tumor core, thus preventing immune infiltration.

**Conclusions:**

Our work supports the idea that distinct spatial locations dictate different CAF transcriptional programs. Targeting specific functional niches will ultimately hinder tumor progression by inhibiting signaling between distinct CAF substates and the surrounding tumor microenvironment.

**Graphical Abstract:**

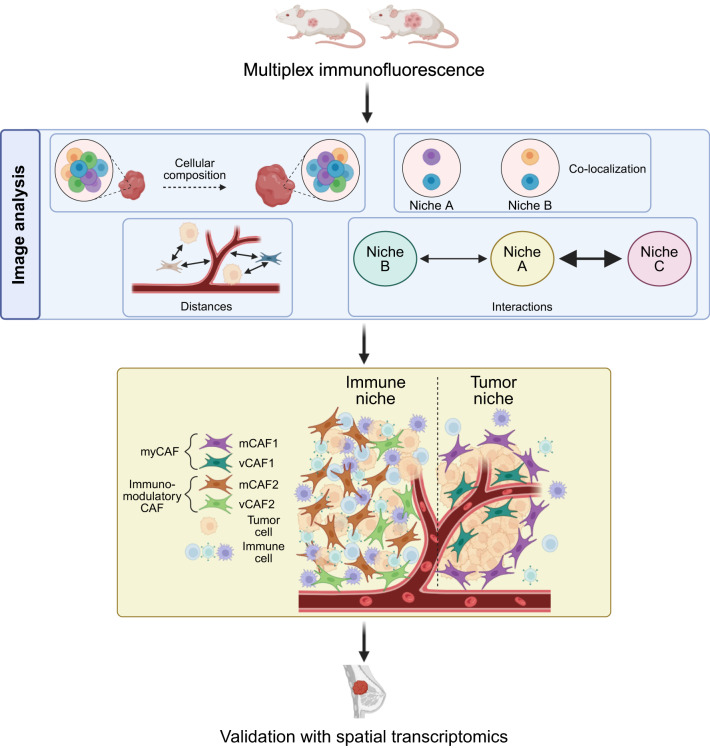

**Supplementary Information:**

The online version contains supplementary material available at 10.1186/s13058-025-02183-7.

## Background

Despite the progress made in cancer treatment, the historical tumor cell-centric view of cancer has neglected the importance of understanding the paracrine signaling within the tumor microenvironment (TME). Here, malignant cells are engaged in paracrine signaling with e.g. immune cells, vascular cells, pericytes, the extra-cellular matrix (ECM) and cancer-associated fibroblasts (CAFs). It is now well known that the TME components act in concert to orchestrate the response to conventional cancer treatments and that exclusive targeting of tumor cells favors the appearance of new (epi)mutations responsible for recurrence and therapy resistance [10]. Thus, to fully understand cancer initiation and progression, it is imperative to consider the tumor as a dynamic organ comprising different cell types that collectively impact patient outcomes.

CAFs are the main constituents of the TME in many solid tumors and the main source for the production of ECM. Notably, multiple hallmarks of cancer are connected to CAF activity [11–13].

In previous work from our group, four subtypes of CAFs were identified through single cell RNA-sequencing (scRNA-seq) in tumors from the MMTV-PyMT transgenic model of breast cancer [1]. The vascular CAFs (vCAFs) are the most abundant and have a gene expression program related to vascular functions; the cycling CAFs (cCAFs) are the proliferating counterpart of the vCAFs; the developmental CAFs (dCAFs) are cancer cells which have undergone an epithelial-to-mesenchymal transition (EMT); finally, the matrix CAFs (mCAFs) are enriched for functions related to extracellular matrix production and immune regulation. These subtypes also have different inferred cellular origins: vCAFs are suggested to originate from the perivascular niche, mCAFs from resident fibroblasts, and dCAFs from cancer cells undergoing EMT. Notably, both mCAFs and vCAFs are associated with a worse prognosis in human breast tumors [1]. The co-existence of distinct CAF subsets is also observed in tumors derived from the triple negative breast cancer (TNBC) cell line 4T1. Here, two distinct subsets are characterized by podoplanin (pCAF) and S100A4 (sCAF) expression, respectively [14]. In human breast cancer, an even higher complexity is observed [15–17]. Moreover, the most comprehensive scRNA-seq dataset on human breast tumor microenvironment has detailed the presence of two main subtypes reminiscent of pancreatic myofibroblastic (myCAF) and inflammatory CAFs (iCAF) [18, 19]. Finally, two perivascular-like (PVL) clusters are described, reminiscent of our previously defined vCAFs in experimental tumors.

In this work, we sought to explore the origin of CAF heterogeneity and delineate how the cellular contexts in which CAFs reside result in distinct functional gene expression programs that determine different CAF substates. To this end, we re-annotated our previously published scRNA-seq dataset at a higher resolution, guiding the development of a multiplex immunofluorescence antibody panel. By using the well-characterized MMTV-PyMT murine model, which develops multifocal mammary neoplasias that progress through well-defined stages that mirror human breast cancer progression, from hyperplasia to metastatic carcinoma [20–22], we interrogated the TME architecture at early and late stages of tumor development and uncovered changes in the TME composition. Spatial analysis of immunostained murine tumor and human spatial transcriptomics revealed a co-localization between immunomodulatory CAFs and immune cells while myofibroblastic CAFs prevented immune infiltration by surrounding the tumor core. Our work supports the potential of precision targeting of specific CAF-related functional niches to hinder tumor progression by, for instance, increasing immune cells infiltration.

## Materials and methods

### Collection of tissue samples

All animal experiments were performed according to institutional guidelines and approved by the local ethics committee in Lund (permit number 14122/2020). Our study exclusively examined female mice since the disease modeled is predominantly relevant in women. Three 8-weeks and three 14-weeks old MMTV-PyMT (FVB/n background strain) female mice were heart-perfused with 10 ml of PBS after injection of 150 mg/kg ketamine (Pfizer #150094) and 5 mg/kg medetomidine (OrionPharma #015602). For paraffin-embedding, tumors were post-fixed in a 10% formalin solution (Sigma-Aldrich #HT501128) overnight before proceeding with dehydration and paraffin embedding.

### Multiplex staining

2,5 μm-thick FFPE sections were deparaffinized and rehydrated through xylene and ethanol solutions. Immunostaining was performed on 11 early-stage and 27 late-stage tumors. Heat-induced epitope retrieval (HIER) was performed with a pressure cooker (2100 Antigen Retriever, BioVendor) in AR6 or AR9 buffer (Akoya Biosciences, #AR600250ML and #AR900250ML). Tissues were stained according to the protocol showed in Table 1 and incubated with an antibody diluent/blocking buffer (Akoya Biosciences, #ARD1001EA) at room temperature. MOTiF Opals were diluted in 1X plus amplification diluent buffer (Akoya Biosciences, #FP1498). Sections were counterstained with DAPI (ThermoFisher Scientific #D3571) and mounted with the ProLong Diamond antifade solution (ThermoFisher Scientific #P36965).

**Table 1:** mIHC panel staining protocol

	Cycle 1	Cycle 2	Cycle 3	Cycle 4	Cycle 5	Cycle 6
HIER	pH9	pH6	pH6	pH6	pH6	pH6
Blocking	30 min	30 min	10 min	20 min	30 min	10 min
Primary antibody and dilution	PDGFRα 1:200	MCAM 1:4000	CD31 1:200	α-SMA 1:1000	CD45 1:800	PanCK 1:200
Manufacturer	Cell signaling #cs3174	Abcam #ab75769	Cell signaling #cs77699	Cell signaling #cs19245	Cell signaling #cs70257	Abcam #ab9377
Incubation (time, temperature)	30 min, RT					
Secondary antibody	Cell signaling #8114S					
Incubation (time, temperature)	10 min, RT	10 min, RT	10 min, RT	30 min, RT	30 min, RT	10 min, RT
OPAL and dilution	Opal 690 1:100	Opal 480 1:100	Opal 520 1:100	Opal 620 1:100	Opal 570 1:100	Opal TSA-DIG 1:100 + Opal 780 1:25
Incubation (time, temperature)	10 min, RT	10 min, RT	10 min, RT	10 min, RT	10 min, RT	10 min + 1 h, RT

### Image acquisition

Whole-slide images were acquired with the PhenoImager HT scanner (Akoya Biosciences) at 20x magnification. The PhenoImager HT 2.0 software (Akoya Biosciences) was used for spectral unmixing and autofluorescence subtraction using the synthetic library as reference. Staining quality was assessed with the Phenochart 2.0 software (Akoya Biosciences) by checking the signal-to-background ratio and Opal normalized counts.

### Image analysis

QuPath v0.5.1 [4] was used for the entire image analysis workflow. Tumor areas, tissue folds and necrotic regions were delineated with the Segment Anything Model (SAM) extension v.0.5.0 [23]. Tissue folds and necrotic regions were excluded from the downstream analysis due to the suboptimal tissue quality.

DAPI-based cell segmentation was performed using the Cellpose 0.9.0 extension running the CP model [4–7]. A random tree machine learning classifier was trained for each marker. MCAM and PDGFRα classifiers were simultaneously applied, while the remaining ones were applied sequentially and hierarchically to avoid unexpected cell phenotypes and as recently recommend [24]. Unclassified cells were excluded from further analysis.

### Neighborhoods identification, clustering and interactions

Cellular coordinates were exported from QuPath for multiplex images and from the Seurat object for the Xenium dataset and then imported in CytoMAP to define 50-µm-radius-neighborhoods with the *Raster Scanned* approach [8]. The FFPE Human infiltrating ductal carcinoma obtained from BioIVT with Custom Add-on Panel-Tissue Sample 1 by 10x Genomics was used for the analysis [25]. Neighborhoods were clustered into regions using a self-organizing map (SOM). Inspection of the Davies-Bouldin function [26] was used to guide the choice of the number of regions that allowed for sufficient clustering resolution. Percentages of shared borders between neighborhood clusters were defined using the *Region Interactions* function in CytoMAP. A 10% and 5% cutoff was used in multiplex images and on the Xenium dataset, respectively, to create chord diagrams with the circlize R package [27] and network plots with the igraph R package [28, 29].

### Measurement of cellular distances

As endothelial cells morphology did not allow an optimal cell segmentation, CD31 intensity threshold was used to establish an intensity-based pixel classifier and to annotate endothelial vessels in QuPath. To mimic endothelial cell segmentation, the resulting vessel annotations were subdivided into 10 µm^2^-tiles. Cellular and tile centroid coordinates were imported into R to measure distances within a 10 μm radius from endothelial tiles, tumor and immune cells using the SPIAT R package [9].

### Immune signatures

Conversion of human gene symbols into murine homologue genes from the Immunome and IRIS databases was performed with Metascape [30].

### Single cell RNA-sequencing analysis

The quality control filtered dataset [1], was normalized, scaled, and clustered (resolution = 0.5) using the Seurat (version 4.0.0) [2] method with default parameters in R (version 4.0.2) [31]. To further investigate the mCAFs at high resolution, we subseted the mCAF population and made a separate subclustering (resolution 0.5) of this subgroup of CAFs. Clusters were visualized with the Uniform Manifold Approximation and Projection (UMAP) method using the SCpubr package (version 2.0.2) [32]. Seurat´s *AddModuleScore* function was used to calculate module scores of iCAF, myCAF and progenitor-like normal fibroblast gene sets (Additional File 7), which all had been collected from tables of the original publications [18, 33, 34]. The module scores were visualized in heatmaps using the *do_EnrichmentHeatmap* function as provided in the SCpubr package. To assess differentiation potency and state of the cells, the CytoTRACE2 package (version 1.0.0) [3] was employed using default parameters.

The canonical pericyte (cell ontology term: CL_0000669) and vascular smooth muscle cell (VSMC, cell ontology term: CL_0000359) marker genes were obtained from the Chan Zuckerberg CELL by GENE Discover CellGuide resource [35]. All canonical marker genes of each cell type were used except for two genes (*Acta2* and *Des*) that were found in both cell type marker lists. The average expression of the canonical marker genes was calculated using the *AverageExpression* function in Seurat and plotted in a heatmap using the *dittoHeatmap* function of the dittoSeq package [36] (version 1.9.0).

Inference of cell-cell communication was performed using the CellChat package (version 2.1.2) and considering only cell-cell contact interactions [37, 38].

### Statistical analysis

Statistical analysis and graphs were generated using ggpubr R package [39].

## Results

### High resolution analysis of scRNA-seq dataset identifies distinct CAF substates

**Fig. 1:**
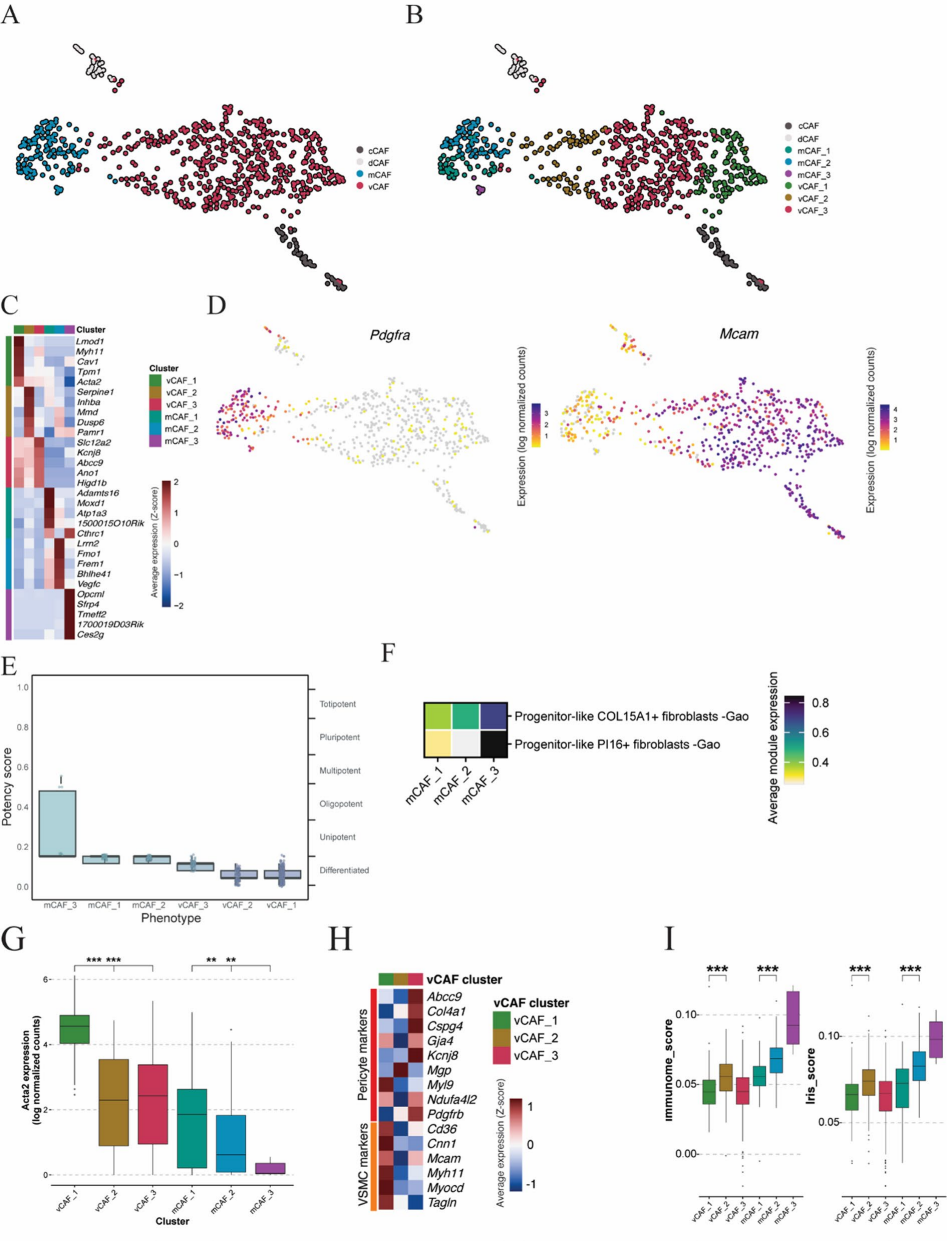
scRNA-seq identifies functionally distinct CAF substates. **A** Uniform manifold approximation and projection (UMAP) visualization of CAF clusters using our originally published scRNA-seq dataset annotation [1]. **B** UMAP visualization with the CAF annotations at higher resolution.** C** Heatmap of top 5 differentially expressed genes (DEGs). Wilcoxon rank sum test was applied to identify DEGs.** D** Pdgfrα and Mcam gene expression plots on UMAP layout.** E** Boxplot of CytoTRACE2 potency score. **F** Heatmap of signatures expression levels of progenitor-like fibroblasts from Gao et al.[34].** G** Boxplot of Acta2 gene expression levels. Wilcoxon test, **p ≤ 0.01, ***p ≤ 0.001.** H** Gene expression heatmap of pericyte and vascular smooth muscle cells (VSMC) genes in the vCAF substates.** I** Boxplot of Immunome and IRIS immune-related signatures expression levels. Wilcoxon test, **p ≤ 0.01, ***p ≤ 0.001

In a previous study, we detailed the heterogeneity of CAFs in the transgenic MMTV-PyMT mouse model of breast cancer [1]. To move beyond this first classification, we hypothesized that CAFs are influenced by paracrine signaling from surrounding cell types (e.g. tumor cells, endothelial cells, and immune cells) or physiological conditions, resulting in the establishment of different CAF substates. To test this hypothesis, we reanalyzed our previously published scRNA-seq dataset at a higher resolution. This was made possible due to the use of the Smart-Seq2 platform for scRNA-seq, which enables more sensitive cluster annotation due to full-length transcript sequencing and high-fidelity alignment [40]. Using the whole dataset (Fig. 1A) and a bioinformatically refined analysis pipeline, the vCAF population was further divisible into three distinct cellular states, whereas none of the other previously defined subgroups, cCAF, dCAF, or mCAF, unveiled any new discrete states (Additional File 1, 

Fig. A). Given that recent studies have revealed robust subtypes of mCAFs [18, 19, 33], i.e. myCAFs and iCAFs, we separated and subclustered the mCAF population to probe this subset in more depth. We identified two major clusters (mCAF1 and mCAF2) plus a small cluster (mCAF3) after re-clustering of the mCAF subset (Additional File 1, Fig. B). In summary, the attempt at refining our previously proposed subdivision of CAFs uncovered three new substates each of vCAFs and mCAFs (Fig. 1B).

To characterize the distinct transcriptional programs of the identified CAF substates, we extracted the differentially expressed genes (DEGs) for each cluster relative to all other clusters (Fig. 1C). mCAF substates were characterized by the uniform expression of *Pdgfra*, whereas all vCAF substates differentially expressed *Mcam* (Fig. 1D).

Given the different reported CAF origins [11, 41], we sought to infer a CAF progenitor state within our dataset. We therefore assessed CAF differentiation states by using the CytoTRACE 2 R package [3]. Compared with mCAFs, vCAFs were more differentiated. The highest potency was observed within the mCAF3 substate (Fig. 1E). This population expressed the highest levels of *Pi16*,* Dpp4*,* Ly6c1*,* Dpt*, *Cd34*, and *Ly6a*, known markers of progenitor fibroblasts [34, 42, 43] (Additional File 1, Fig. C). Moreover, gene signatures of the fibroblast progenitor clusters identified by Gao et al., [34]. were higher expressed by mCAF3 (Fig. 1F). Therefore, we concluded that mCAF3 represented the substate of origin for mCAFs.

The known fibroblast marker *Acta2*, encoding for α-SMA (α-smooth muscle actin), was significantly higher expressed by vCAF1 and mCAF1, indicating the presence of two distinct myofibroblastic states (Fig. 1G). vCAF1 was further characterized by the expression of vascular smooth muscle cell-typical genes, such as *Myh11* and *Tagln* (Fig. 1H). Additionally, mCAF1 exhibited high expression levels of *Lrrc15*, a recently described myofibroblast marker (Additional File 1, Fig. D) [34, 42, 44, 45].

The most abundant vCAF substate, vCAF3, was characterized by intermediate *Acta2* expression levels (Fig. 1G) and by the differential expression of markers typical of pericytes, such as *Abcc9* and *Kcnj8* (Fig. 1H). Thus, vCAF3 had a pericyte-like profile, possibly indicating a pericyte-to-fibroblast transition state.

Further, to assess the crosstalk between the CAF substates and the immune system, we used the Immunome [46] and IRIS [47] human databases to generate immune-gene signatures. Upon conversion into the corresponding murine homologue genes, both the Immunome and the IRIS immune signatures were significantly higher expressed by vCAF2 and mCAF2, compared to vCAF1 and mCAF1 (Fig. 1I). Notably, the mCAF3 substate had the highest immune score. Collectively, these data suggest that vCAF2 and mCAF2 bear immunomodulatory properties, and that *Acta2* expression is negatively correlated with such CAF-related immune functions. Supporting our observations, two previously defined iCAF signatures were higher expressed by mCAF2, whereas two published myCAF signatures were expressed at the highest levels by mCAF1 (Additional File 1, Fig. E) [18, 33].

Taken together, the mCAF and vCAF subtypes could be further divided into three substates, with a myofibroblast and an immunomodulatory state observed within each CAF subtype.

### Multiplex immunofluorescence and spatial analysis validate the presence of spatially segregated CAF substates

**Fig. 2:**
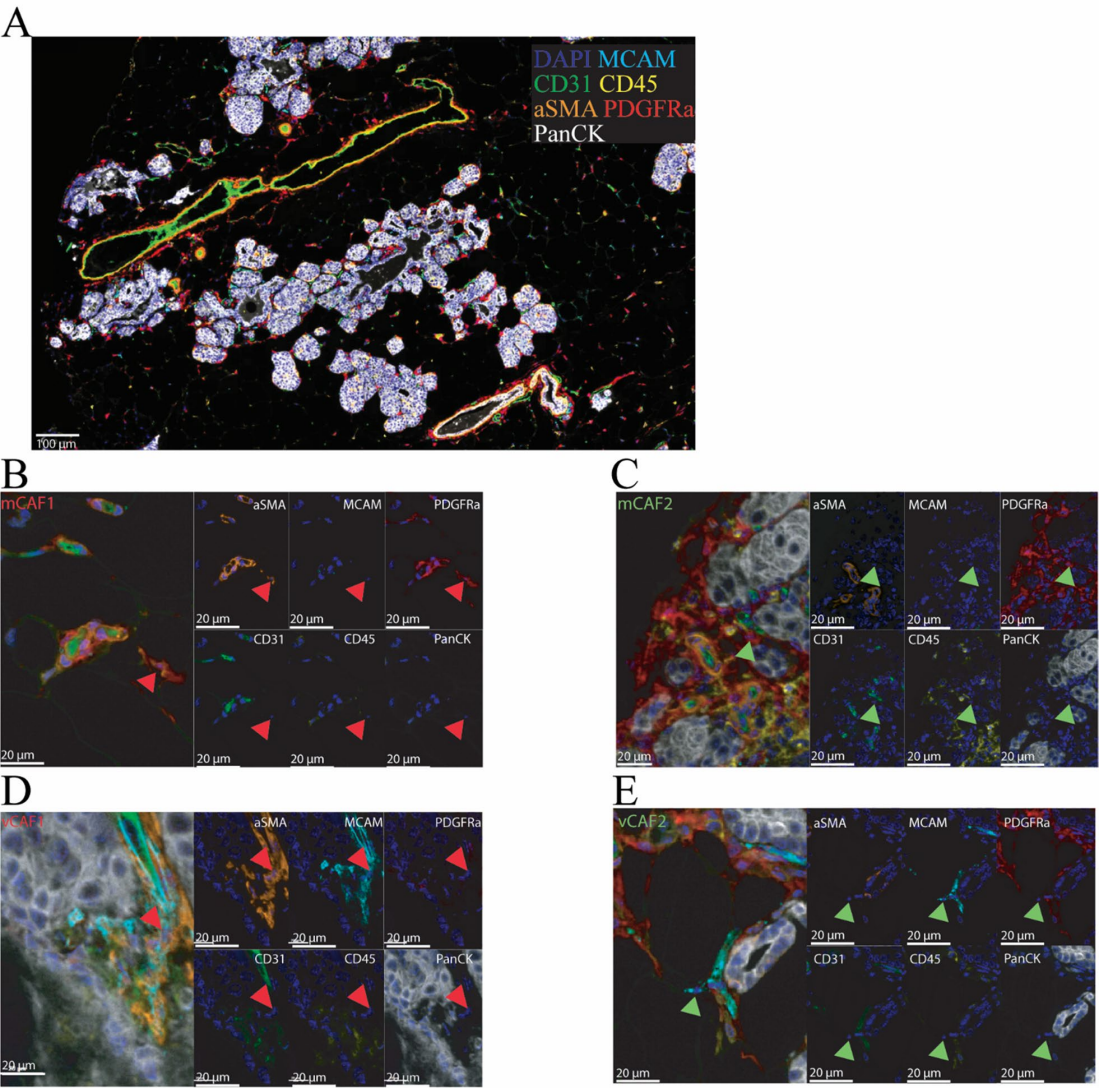
Multiplex immunofluorescence identifies distinct CAF substates.** A** Representative mIHC image of an early-stage MMTV-PyMT tumor. **B**–**E** Identification of CAF substates of interest based on the staining intensity of each marker in their relative single channel output. Red arrows indicate mCAF1 (α-SMA+/PDGFRα+, B) and vCAF1 (α-SMA+/MCAM+, D), while green arrows mCAF2 (α-SMA-/PDGFRα+, C) and vCAF2 (α-SMA-/MCAM+, E)

**Fig. 3:**
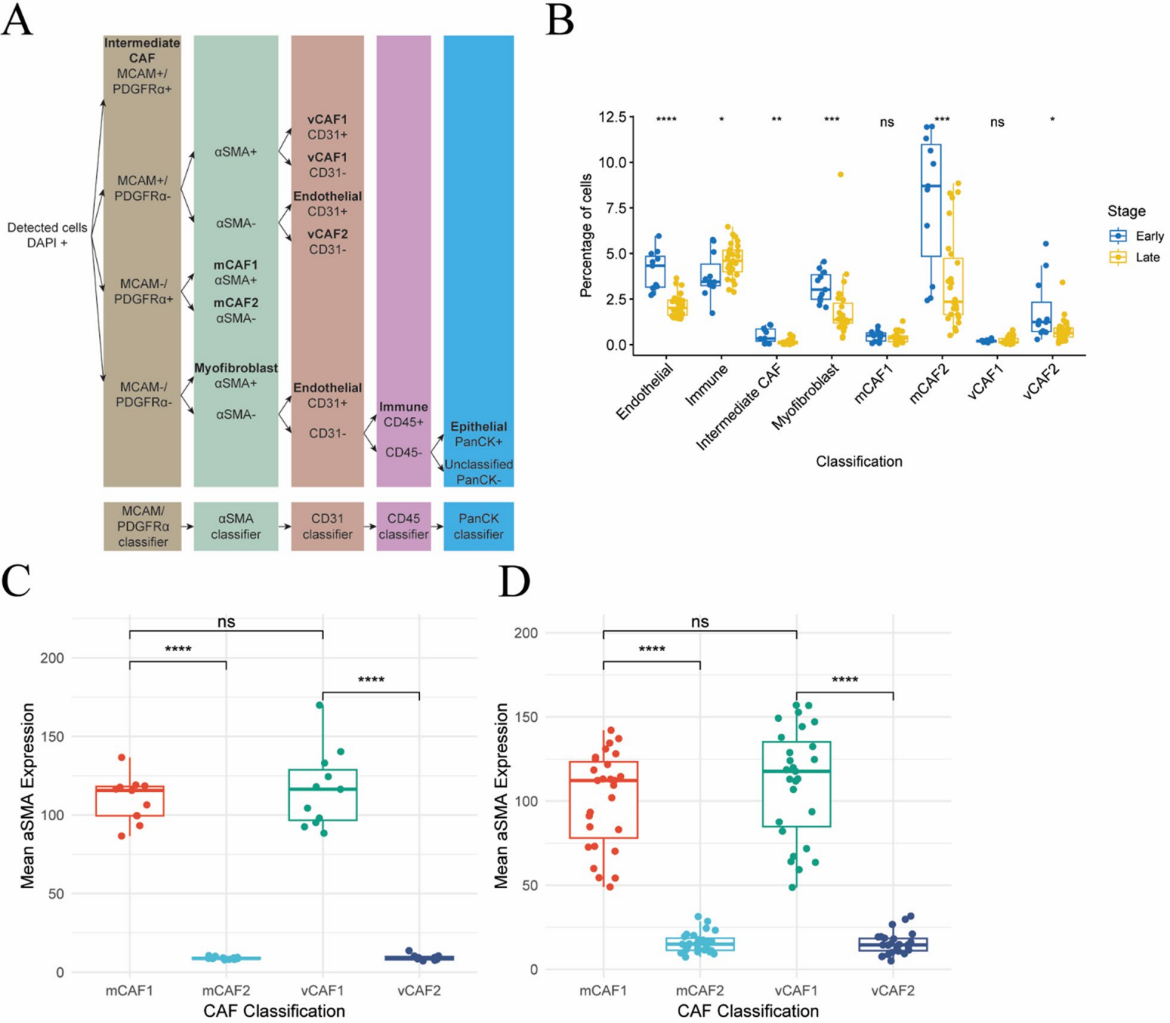
Image analysis identifies changes in stromal composition across tumor progression.** A** QuPath phenotyping scheme based on sequentially applied machine learning classifiers.** B** Percentages of stromal cells in early- and late-stage tumors. **C**–**D** Mean CAF α-SMA immunostaining intensity in early-stage (**C**) and in late-stage tumors (**D**). N = 11 early-stage and n = 27 in late-stage tumors. Wilcoxon test, *p ≤ 0.05, **p ≤ 0.01, ***p ≤ 0.001, ****p ≤ 0.0001, ns =* p* >0.05

Based on the delineation of CAF substates, we hypothesized that the distinct gene expression programs are dictated by different spatial locations. To test this hypothesis, we exploited multiplex immunohistochemistry (mIHC) by staining tumors from MMTV-PyMT mice collected at 8 weeks of age (neoplasia stage; 11 tumors from 3 mice) and at 14 weeks of age (late carcinoma stage; 27 tumors from 3 mice). These time points were selected to capture the transition from intraepithelial neoplasia to invasive carcinoma, reflecting the progression observed in human ductal breast cancer [20–22]. Tissues were immunostained for MCAM (to mark all vCAFs), PDGFRα (mCAFs), α-SMA (myofibroblasts and myofibroblastic CAFs), CD31 (endothelial cells), CD45 (immune cells) and pan-cytokeratin (CK) (tumor epithelial cells) (Fig. 2A and S2). To perform cell segmentation, we used the Cellpose extension [5–7] in QuPath [4]. The mCAF1 state was defined as PDGFRα^+^/α-SMA^+^ (Fig. 2B), the mCAF2 state as PDGFRα^+^/α-SMA^−^ (Fig. 2C), the vCAF1 state as MCAM^+^/α-SMA^+^ (Fig. 2D), and the vCAF2 state as MCAM^+^/α-SMA^−^ (Fig. 2E). Trained machine-learning classifiers were established for each marker and then combined sequentially to identify the cell phenotypes of interest (Fig. 3A, Additional File 2). As we wanted to focus our study on immunomodulatory and myofibroblastic CAFs, we could not definitively identify mCAF3 and vCAF3 with our phenotyping approach. However, we identified a population of MCAM^+^/PDGFRα^+^ CAFs, here termed intermediate CAFs, likely containing the mCAF3 and vCAF3 substates (Fig. 3A–B).

In agreement with our scRNA-seq data, α-SMA intensity was significantly higher in mCAF1 and vCAF1 compared with mCAF2 and vCAF2, respectively (Fig. 3C–D). Interestingly, the expression of *Acta2* was higher for vCAF1 compared with mCAF1 in the scRNA-seq data analysis (Fig. 1G), but we could not observe this difference at the protein level. This observation suggests that *Acta2* expression levels are regulated post-transcriptionally in CAFs and that this effect might be due to distinct CAF spatial locations.

To identify differences in TME composition, cell counts revealed that, on the one hand, the percentage of immune cells significantly increased upon tumor progression from 8 to 14 weeks of age of MMTV-PyMT mice (Fig. 3B). On the other hand, late-stage tumors showed a decreased overall proportion of mCAFs and vCAFs, myofibroblasts and endothelial cells (Additional File 3, Fig. A). When inspecting the mCAF and vCAF populations, the abundance of myofibroblastic mCAF1 and vCAF1 was not significantly different over time (Fig. 3B). However, both the immunomodulatory mCAF2 and vCAF2 states were significantly reduced during tumor development (Fig. 3B). Interestingly, and in contrast to our scRNA-seq dataset, the proportion of mCAFs was higher compared to the one of vCAFs at both time points, illustrating the need for caution when inferring cellular abundance from scRNA-seq data (Additional File 3, Fig. A).

**Fig. 4:**
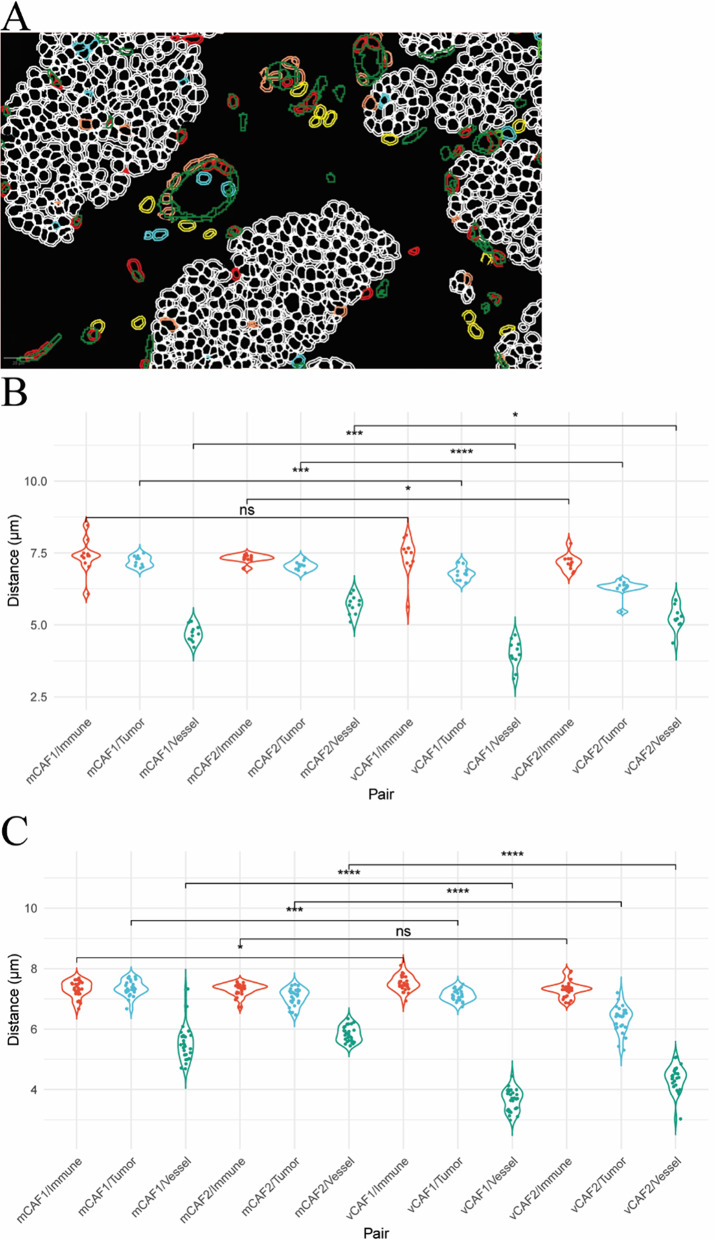
Image analysis identifies distinct CAF locations.** A** Representative image of Cellpose cell segmentation with applied phenotyping scheme and vessel tiles. vCAF1 in blue, vCAF2 in cyan, mCAF2 in red, endothelial cells and tiles in green, myofibroblasts in orange, immune cells in yellow, and tumor cells in white. **B**–**C** Mean distances (µm) from CAFs to the closest immune, tumor cell, and vessel tiles in early (**B**) and late-stage (**C**) tumors. N = 11 early-stage and n = 27 in late-stage tumors. Wilcoxon test, *p ≤ 0.05, **p ≤ 0.01, ***p ≤ 0.001, ****p ≤ 0.0001, ns =* p* >0.05

Next, to delineate distinct cellular localizations, we measured distances from CAFs to vessels, immune cells and tumor cell centroids using the SPIAT R package [9]. To overcome the limitation of poor cell segmentation of endothelial vessels, we used a CD31 pixel thresholder to annotate endothelial vessels which were further divided into 10 µm^2^ tiles as proxies for cells (Fig. 4A). Considering a radius of 10 μm, vCAFs were significantly closer to vessels and tumor cells, compared with mCAFs (Fig. 4B–C). When considering a radius of 100 μm in early-stage tumors, vCAF distances to vessels, and in particular from vCAF2, had a bimodal distribution (Additional File 3, Fig. B). Moreover, the closer proximity of vCAF1 to vessels in the late-stage tumors, compared with vCAF2 (Fig. 4C), suggests that vCAF1 are retained near endothelial vessels, indicating a potential mural cell function for this vCAF substate, whereas vCAF2 leave the peri-vascular space to reside in distant locations. In summary, given the closer vCAF proximities to other cellular elements compared with the mCAFs, these data suggest that vCAFs may constitute a paracrine hub for interactions within the TME and support our previous observation of a peri-vascular origin of vCAFs.

### Identification of distinct cellular neighborhoods and spatial segregations of CAF substates

**Fig. 5:**
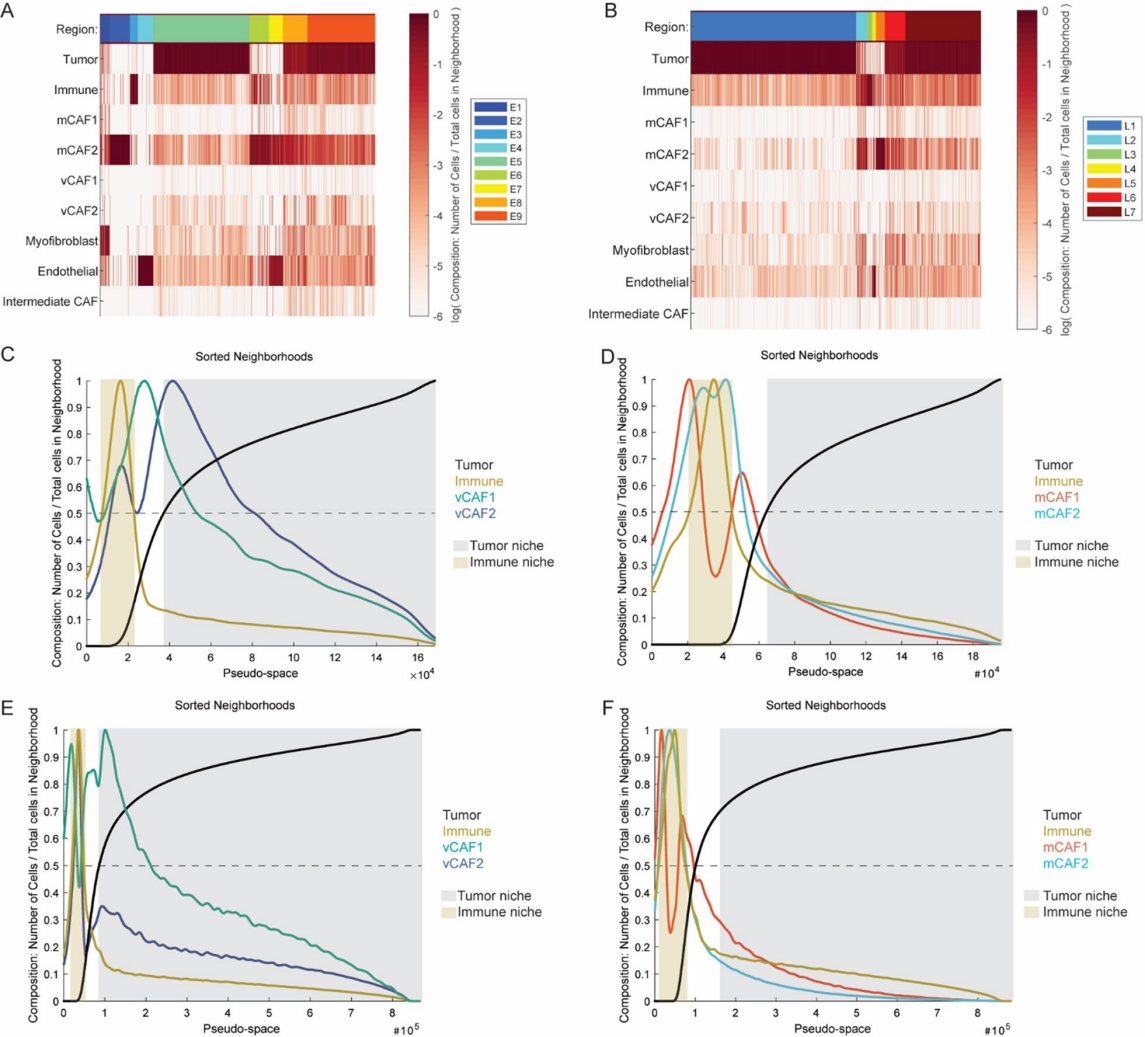
Spatial analysis identifies distinct cellular neighborhoods and CAF substate locations.** A** Heatmap of early-stage neighborhood cellular compositions clustered into 9 regions.** B** Heatmap of late-stage neighborhood cellular compositions clustered into 7 regions. **C**–**F** Pseudo-space plots with the neighborhoods sorted based on immune cell and CAF composition in early- (**C**–**D**) and late-stage tumors (**E**–**F**). N = 11 early-stage and n = 27 in late-stage tumors.

To study the tumor cellular organization we conducted a hierarchical spatial analysis (Additional File 4, Fig. A). Firstly, we defined 50-µm-radius neighborhoods using the *raster scan neighborhoods* function in CytoMAP [8]. A self-organizing map (SOM) clustered the neighborhoods into distinct tissue regions. Nine regions were identified in early-stage tumors (E1-9) and seven in late-stage samples (L1-7) (Fig. 5A–B and Additional File 4, Fig. B and C). The identified regions were evenly represented in all early-stage tumors, while all regions were present in nearly all late-stage samples (Additional File 4, Fig. D and E).

Inspection of the cellular composition of each region (Additional File 5, Fig. A and B) revealed the presence of three malignant cell-enriched regions in both early-stage lesions (E5, E9, E8) and in late-stage lesions (L1, L7, L6). Immune cells were exclusively enriched in one region at each tumor stage (E3 and L3). Similarly, mCAF2 were exclusively enriched in regions E2 and L5. Interestingly, regions E6 and L2 were enriched for both mCAF2 and immune cells, representing mixed regions where both cell types were present in high proportion. Thus, these data suggest a spatial proximity between immune cells and the immunomodulatory mCAF2. Endothelial cells were enriched in two clusters at early-stage (E4 and E7) but only in one at 14 weeks (L4). Finally, only in early-stage tumors, we observed the presence of a distinct region (E1) enriched with myofibroblasts (Additional File 5, Fig. A). These observations were in accordance with the previously observed reduction in myofibroblasts and endothelial cell contents in late-stage tumors (Fig. 3B).

Whereas it was not possible to directly compare cell percentages between regions given the different CAF cellular densities and region extensions, we explored pseudo-space analysis to investigate CAF preferential localizations within the 50-µm-radius neighborhoods. By sorting the neighborhoods based on their cellular compositions, the pseudo-space analysis revealed that, in early lesions, neighborhoods enriched with immune cells contained more vCAF1 than vCAF2, and more mCAF2 than mCAF1. Interestingly, neighborhoods showing the highest concentration in immune cells had a strong reduction in mCAF1 content. On the contrary, neighborhoods enriched with tumor cells contained more vCAF2 than vCAF1, and slightly more mCAF2 than mCAF1 (Fig. 5C and D). In summary, these results showed that, at early-stage, vCAF1 and mCAF2 co-localize more with immune cells than tumor cells, while vCAF2 co-localize more with tumor cells than immune cells.

In late-stage tumors, neighborhoods enriched with immune cells contained more vCAF2 than vCAF1, and more mCAF2 than mCAF1. On the contrary, neighborhoods enriched with tumor cells contained more vCAF1 than vCAF2, and more mCAF1 than mCAF2 (Fig. 5E and F). In conclusion, these results showed that, at late-stage, myofibroblastic CAFs co-localized with tumor cells, while immunomodulatory CAFs with immune cells.

Taken together, these observations suggest that the distinct CAF transcriptional programs are dictated by different spatial locations and interactions within 50-µm-radius microniches.

### Neighborhood regions interact to form three spatially defined functional niches

To further delineate the spatial organization of tumors and cellular interactions, we exploited the *Region Interactions* function in CytoMAP [8]. Percentages of shared borders between regions were averaged across tumors (Additional File 5, Fig. C-D) and a 10% cut-off was set up to study cross-regional interactions (Additional File 5, Fig. E–F).

**Fig. 6:**
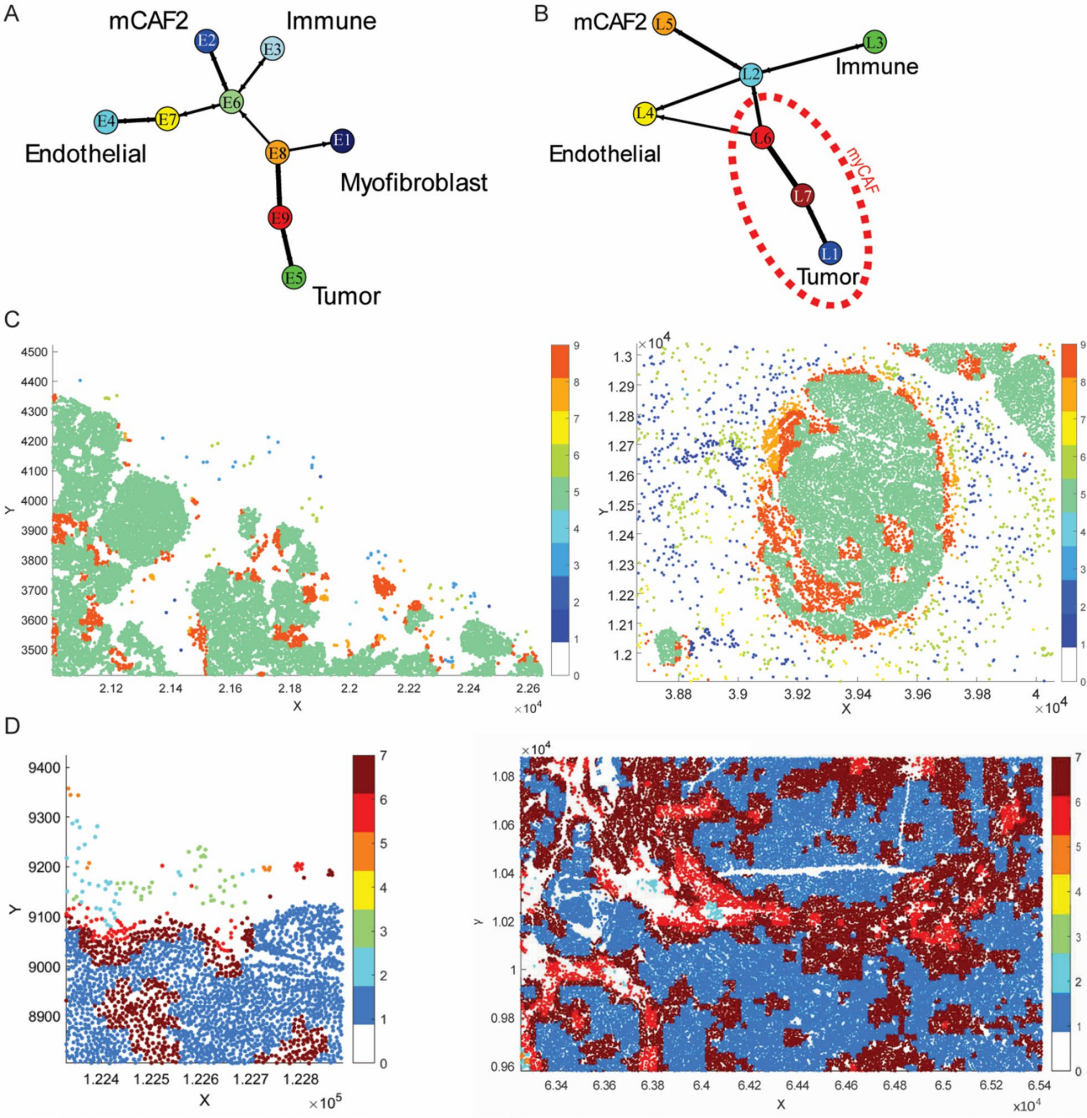
Neighborhood interactions analysis identifies distinct spatially located functional niches and CAF-related enrichment. **A**–**B** Network plots showing the spatial distribution of early- (**A**) and late-stage (**B**) neighborhood clusters. The red-dashed region indicates the area where myofibroblastic CAFs were mainly concentrated. **C**–**D** Region color-coded positional plots of the identified neighborhoods. Representative immune (left), and tumor niche (right) in early- (**C**) and late-stage (**D**) tumors.

In both early and late-stage tumors, tumor cell-enriched regions mainly interacted among themselves. Regions enriched individually with immunomodulatory mCAF2 (E2 and L5) and immune cells (E3 and L3) interacted with a region that was enriched with both cell types (E6 and L2) (Fig. 6A–B). These results indicate paracrine signaling and further support spatial proximity between immune cells and immunomodulatory mCAF2.

The interactions described uncovered the existence of three main functional niches: tumor, immune and endothelial. Considering the spatial relationships between regions and the previously observed co-localizations within 50-µm-radius neighborhoods, we concluded that, in late-stage tumors, myofibroblastic CAFs surrounded the neighborhoods enriched with tumor cells (Fig. 6B). Notably, in both early- and late-stage tumors, mCAF2 and immune-enriched regions did not share borders with tumor-enriched regions (Fig. 6A–B). This result is in accordance with our previous observations indicating that mCAF2 and immune cells spatially co-localize (at late-stage) and that they are excluded from tumor-enriched niches. Of note, the model suggested that myofibroblastic CAF-enriched regions were localized as a barrier between mCAF2/immune cell niches and malignant cells. Taken together, our findings suggest distinct spatial locations for the identified functional cellular niches where different CAF substates were enriched. In particular, myofibroblastic CAFs appeared to be more concentrated around the tumor niche in late-stage tumors, while the immunomodulatory CAFs reside together with infiltrating immune cells.

Visual inspection of positional plots validated our identified spatial architecture. At both time points, the tumor-enriched regions displayed a concentric organization, with the highest density of tumor cells located at the center of the tumor core (right panels in Fig. 6C–D). Additionally, the spatial proximity observed between immune cell-exclusive regions (E3 and L3), mCAF2-exclusive regions (E2 and L5), and mixed regions (E6 and L2) further reinforced the spatial association between immune cells and immunomodulatory mCAF2 (left panels in Fig. 6C–D).

### Spatial analysis of human dataset recapitulates murine tumor architecture

**Fig. 7:**
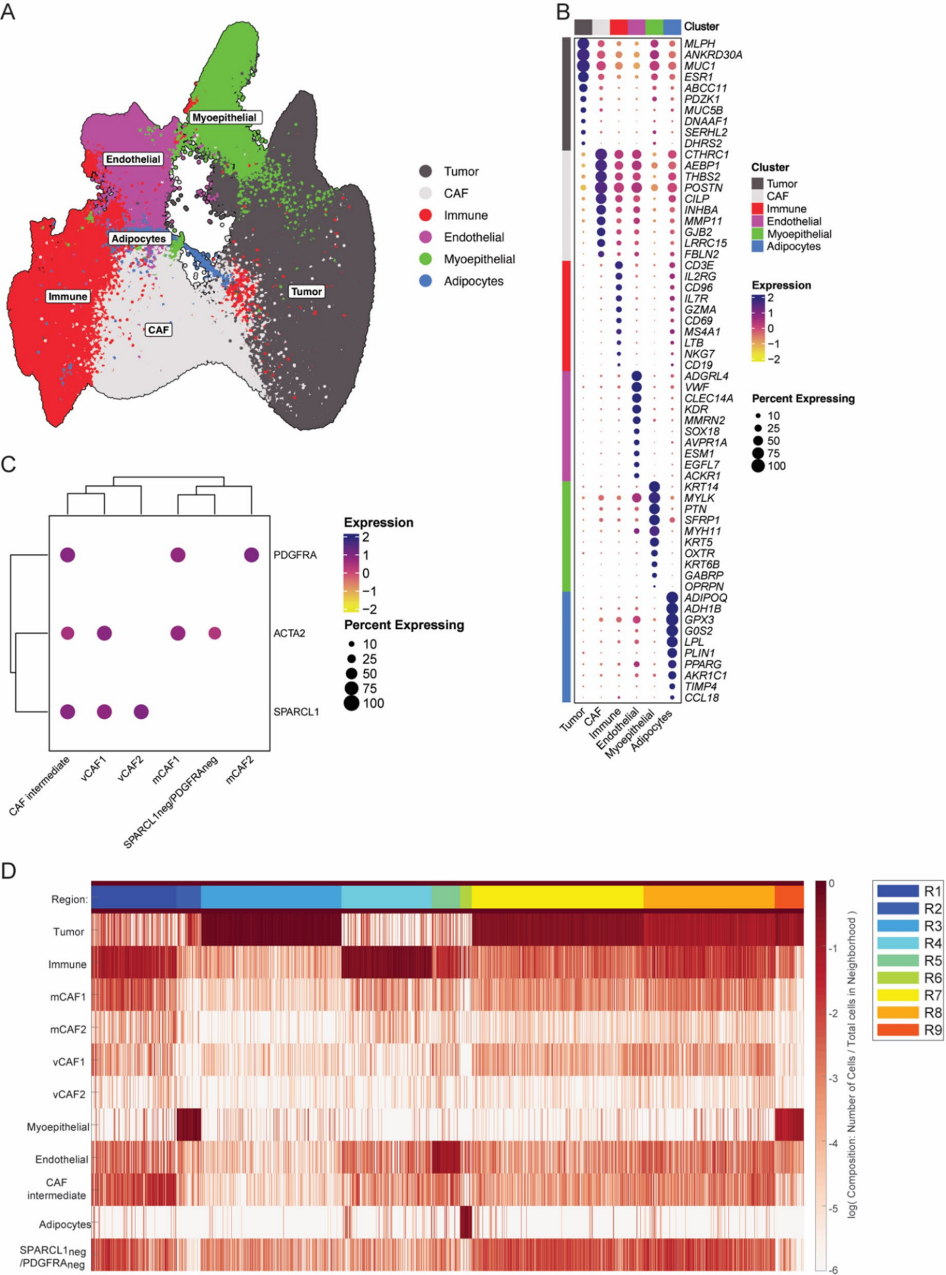
Identification of CAF substates and functional niches in human Xenium panel.** A** UMAP of identified Xenium clusters.** B** Clustered dot plot of top 10 DEGs between cell types.** C** Clustered dot plot of SPARCL1, ACTA2 and PDGFRα expression in the re-annotated Xenium dataset.** D** Heatmap of neighborhood cellular compositions clustered into 9 regions

As our data pointed to a retained presence of the CAF substates across species, we set out to validate our observations in a publicly available spatial transcriptomics dataset from a human breast cancer sample analyzed with the 10X Genomics Xenium technology. To achieve this goal, we reannotated the dataset and identified the CAF substates using *PDGFRA* (pan-mCAF), *ACTA2* (mCAF1 and vCAF1) and *SPARCL1* probes (pan-vCAF; *MCAM* was not available in the gene panel) (Fig. 7A–C and S6A). Spatial coordinates were extracted and imported into CytoMAP to perform the spatial analysis. Neighborhood clustering identified nine regions (Fig. 7D and S6B–C). We observed the presence of three main tumor regions (R3, R7, and R9), an immune-enriched (R4), an adipocyte-enriched (R6), an endothelial-enriched (R5), and a myoepithelial-enriched region (R2) (Fig. 7D and Additional File 6, Fig. D).

**Fig. 8:**
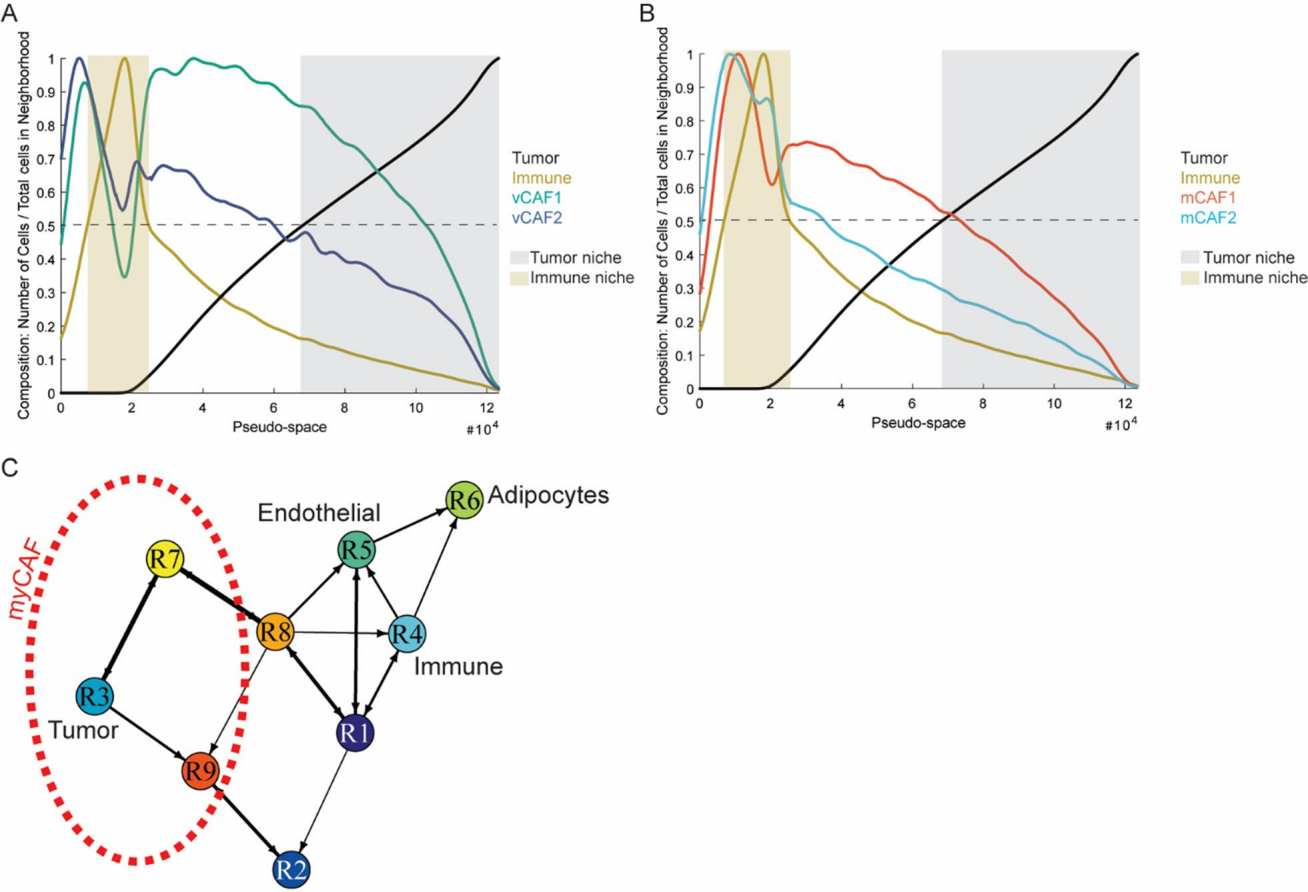
Validation in human Xenium panel recapitulates the spatial organization observed in murine tumors. **A**–**B** Pseudo-space plots with the neighborhoods sorted based on immune cell and CAF composition. **C** Network plot showing the spatial distribution of neighborhood clusters. The red-dashed region indicates the area where myofibroblastic CAFs were mainly concentrated

Pseudo-space analysis showed that neighborhoods enriched with immune cells contained more vCAF2 than vCAF1, and more mCAF2 than mCAF1. On the contrary, neighborhoods enriched with tumor cells contained more vCAF1 than vCAF2, and more mCAF1 than mCAF2 (Fig. 8A–B). In summary, these results confirmed the spatial co-localization between immune cells and immunomodulatory CAFs, and tumor cells and myofibroblastic CAFs observed in the MMTV-PyMT mouse model.

Analysis of region interactions, using a 5% cut-off of sharing borders between regions, revealed the presence of different functional niches exhibiting distinct spatial locations and interactions (Fig. 8C and Additional File 6, Fig. S6E). As observed in the experimental murine tumors, regions enriched with tumor cells (R3, R7 and R9) strongly communicated with themselves and did not directly interact with the most immune-enriched regions (R4 and R1). Finally, considering the previously observed co-localizations within neighborhoods, we confirmed that myofibroblastic CAFs surrounded the neighborhoods enriched with tumor cells.

To further characterize the immune context of immunomodulatory CAFs in human breast cancer, we performed gene expression analysis of the regions most enriched for iCAFs in the Xenium dataset analyzed here. We found elevated expression of CD3E, CD4, CD8A, CD68, and MS4A1 in conjunction with immunomodulatory CAF species, indicating the presence of T cells, macrophages, and B cells (Fig. S6F). In agreement, we performed CellChat analysis on a comprehensive scRNA-seq dataset of human breast cancers [18, 37, 38], which revealed that iCAFs interact predominantly with CD8⁺ T cells and cycling T cells (Fig. S6G).

Taken together, the spatial transcriptomic analysis of a human breast cancer supported the analysis of the tumor architecture of murine mammary carcinomas from MMTV-PyMT mice, demonstrating that myofibroblastic CAFs co-localized with tumor cells and shielded them from immune cell-enriched niches co-inhabited by immunomodulatory CAF substates.

## Discussion

Here, we have identified six CAF substates by analyzing our previously published scRNA-seq data of CAFs from the MMTV-PyMT model of breast cancer. Two substates were characterized by an immunomodulatory gene expression program (mCAF2 and vCAF2), two other substates exhibited a myofibroblast-like profile (mCAF1 and vCAF1), while mCAF3 harbored fibroblast progenitor-like potential, and vCAF3 were defined as pericyte-like CAFs. Notably, the immunomodulatory and myofibroblast-like gene expression profiles were mutually exclusive as *Acta2* (encoding for α-SMA) expression levels were higher in the myofibroblastic CAFs and lower in the immunomodulatory CAFs. Immunostaining of experimental breast cancers at different stages of tumor progression revealed a higher immune cell content in late-stage tumors that was coupled with a decrease in the number of immunomodulatory CAFs. Spatial analysis of murine and human tumor architecture revealed the presence of functional niches where distinct CAF substates were enriched. In particular, we observed that myofibroblastic CAFs were positioned in proximity to malignant cell-enriched regions between the tumor cells and niches composed of the immunomodulatory mCAF2 together with immune cells. Thus, we infer that distinct CAF transcriptional programs may be shaped by their interactions with different cell types within specific functional niches.

CAFs have been described to exert both pro- and anti-tumorigenic functions due to their transcriptional heterogeneity and cellular origins. Several studies have dissected CAF heterogeneity in both human and murine breast cancer at the single-cell resolution, uncovering different subtypes and substates. Specifically, FAP^hi^ α-SMA^hi^ CAF-S1 have been shown to be located close to tumor cells in human breast cancer exerting an immunosuppressive function. On the other hand, FAP^neg^ α-SMA^hi^ CAF-S4 co-expressed MCAM but were not found to be close to tumor cells [15]. CAF-S1 were further subdivided into three subclusters belonging to the inflammatory (iCAF) subgroup, and five belonging to the myofibroblastic (myCAF) subgroup. Notably, the abundance of two myCAF subclusters (ecm-myCAF and TGFβ-myCAF) was correlated with an immunosuppressive environment and primary resistance to immunotherapy in melanoma and non-small-cell lung cancer (NSCLC) patients [16]. In accordance with these observations, we showed that myofibroblastic CAFs were enriched in regions co-inhabited by tumor cells, thus supporting their spatial proximity. Our spatial analysis showed that myofibroblastic CAFs sustained an immunosuppressive role by shielding the tumor core preventing immune cell infiltration. Additionally, our observation was extended also to MCAM-expressing vCAF1. Further, *Lrrc15* has been shown to be expressed by myofibroblasts, connected to CD8^+^ T cells suppression, and limited responsiveness to checkpoint blockade [34, 42, 44, 45]. In accordance with these findings, *Lrrc15* was highly expressed by the mCAF1 substate. Comprehensive single-cell atlases of human breast cancer further confirmed the existence of iCAFs and myofibroblastic CAFs [17, 18] reminiscent of the mCAF1 and mCAF2 substates, respectively. Two studies in pancreatic cancer have shown the coexistence of myCAFs and iCAFs [19, 33], thereby underpinning the generalization of our current work. In accordance with our results, myCAFs were closer to neoplastic cells while iCAFs were more distant and did not have high α-SMA expression levels [19].

Albeit small in prevalence, the mCAF3 subset emerged as a progenitor state for the mCAFs. In keeping with this, mCAF3 expressed the highest levels of a gene signature for universal fibroblast progenitors and stemness-associated genes (*Cd34* and *Ly6a*) [34, 42, 48]. Taken together, these findings suggest that mCAFs originate from normal residential fibroblasts. Moreover, we observed that mCAF3 expressed the highest immune score and the highest expression levels of *Cxcl12*, a known iCAF marker [17, 33, 48], as such confirming the previously identified association between Pi16^+^ fibroblasts and increased pro-inflammatory signaling [34]. Despite the small cell number, we considered this a robust and biologically meaningful cluster, as the dataset was generated using the Smart-seq2 platform, which provides full-length transcript coverage and high sensitivity, enabling the reliable identification of rare cell populations [49]. Finally, vCAF3 were reminiscent of pericytes, had intermediate *Acta2* expression levels and the highest potency score among vCAFs, thus hinting to a lineage relationship with the other vCAF substates.

Due to the limited number of CAF-specific antibodies contained in our mIHC panel, we were not able to fully translate our scRNA-seq findings at the protein level. However, we observed the presence of MCAM^+^/PDGFRα^+^ CAFs (corresponding to the SPARCL1^+^/PDGFRα^+^ CAFs in the Xenium dataset), potentially containing other reported CAF subtypes (e.g., antigen-presenting CAFs) [14, 17, 33]. To further characterize the spatial location of these and other CAF subtypes, future studies should employ higher-resolution mIHC and custom-designed spatial transcriptomics panels. These approaches will enable a more detailed and accurate mapping of CAF heterogeneity within the TME.

Taken together, our work is consistent with the hypothesis that distinct CAF substate gene expression profiles are dictated by different spatial locations. We infer that tumor and immune cells actively instruct CAFs, shaping their transcriptional profiles and dictating the spatial architecture we observed in late-stage experimental tumors and in human breast cancer. Indeed, it has been previously reported that cancer cells can activate *Acta2* expression via the TGF-β axis [50, 51]. In the 4T1 mouse breast cancer model, the LATS1/2 kinases expressed by tumor cells increased the proportion of NCAM1^+^ α-SMA^+^ CAFs, which were associated with an immunosuppressive TME and expressed TGF-β [52]. On the other hand, CAFs have been shown to form a capsule that enwraps cancer cells restricting colorectal tumor growth [53]. These observations suggest that CAFs may have different roles at different stages of tumor development, and that their function is influenced by signaling coming from the local architecture they are embedded in. Indeed, previous studies have demonstrated that CAF transcriptional programs change over time [14], pointing out the dynamic regulation of CAF substates.

Understanding how CAF substate and niche dynamics contribute to tumor initiation and progression is thus fundamental to improve the precision of cancer treatments. By uncovering this aspect, our longitudinal study proved a progressive increase in immune cell content that was concomitant with a reduction in endothelial cells, myofibroblasts and immunomodulatory CAFs. Of note, the abundance of both CD4^+^ T cells and F4/80^+^ macrophages has been reported to increase during tumor progression in the MMTV-PyMT model [54]. Our study was not designed to distinguish between different types of immune cells; future work will aim to elucidate the co-existence of CAF substates with more specialized immune entities. However, our spatial transcriptomic data suggests the presence of T cells, macrophages, and B cells in regions enriched for iCAFs, based on elevated expression of CD3E, CD4, CD8A, CD68, and MS4A1. Complementarily, CellChat analysis revealed strong inferred communication between iCAFs and CD8 + T cells and cycling T cells. Together, these data support a spatial and functional association between immunomodulatory CAFs and specific immune subsets. Moreover, and in accordance with the work presented here, mCAF2 were also reduced in late-stage tumors from MMTV-PyMT in the C57BL/6 strain background [55]. Whereas we quantified a decrease in the number of vCAFs, particularly of vCAF2, in the MMTV-PyMT tumors from FVB/N hosts used in this report, vCAFs were reported to increase during tumor progression in the C57BL/6 strain. This discrepancy might root back to the different reported kinetics of the PyMT tumorigenesis in these two strains [20, 56]. The shorter distance between vCAFs and endothelial vessels compared with mCAFs support their suggested peri-vascular origin and a potential role in tumor angiogenesis of vCAFs, especially in early-stage tumors. However, biological differences between the FVB/NJ and C57Bl/6J backgrounds may influence the way tumor and immune cells [57] instruct the vCAF population.

Understanding the complex and dynamic signaling between the different components of the TME is fundamental to develop precision targeting of specific circuitries associated with more aggressive tumor features. These evolving signaling cues have often limited the success of clinical intervention, thereby positioning the targeting of CAF substates and/or paracrine niches as potential therapeutic enablers of traditional tumor cell-targeting drugs or immunotherapies. In this regard, our work paves the way for the conceivable exploitation of specific CAF substates as biomarkers for prognostication or to drive treatment decisions.

## Conclusions

Our study demonstrates that CAF transcriptional programs are shaped by their spatial context and interactions with neighboring cell types within the tumor microenvironment. We identified six CAF substates with distinct gene expression profiles, spatial distributions, and functional roles. Longitudinal analysis revealed a progressive increase in immune cell content during tumor progression, accompanied by a reduction in endothelial cells, myofibroblasts, and immunomodulatory CAFs. Our findings support a model in which evolving paracrine signals and niche-specific interactions instruct CAF identity and function. This spatial and temporal heterogeneity positions CAF substates as promising biomarkers and therapeutic targets. Precision targeting of CAF-related niches may enhance the efficacy of conventional therapies and immunotherapies by reshaping the tumor microenvironment.

## Supplementary Information

Below is the link to the electronic supplementary material.

Supplementary Material 1.

Supplementary Material 2.

Supplementary Material 3.

Supplementary Material 4.

Supplementary Material 5.

Supplementary Material 6.

Supplementary Material 7.

## Data Availability

All data generated in the context of this study is available from the authors upon reasonable request.

## Abbreviations

CAF: Cancer-associated fibroblast
cCAF: cycling CAFs
CD31: Cluster of differentiation 31
CD45: Cluster of differentiation 45
dCAF: developmental CAF
ECM: Extra-cellular matrix
EMT: Epithelial-to-mesenchymal transition
iCAF: Inflammatory cancer-associated fibroblast
mCAF: matrix CAFs
MCAM: Melanoma cell adhesion molecule
MMTV-PyMT: Mouse mammary tumor virus-polyoma middle tumor-antigen
myCAF: Myofibroblastic cancer-associated fibroblast
Pan-CK: Pan-cytokeratin
PDGFRA: Platelet-derived growth factor receptor A
scRNA-Seq: single-cell RNA sequencing
TME: Tumor microenvironment
TNBC: Triple negative breast cancer
UMAP: Unifold Manifold Approximation and Projection
vCAF: vascular CAFs
α-SMA: α-Smooth muscle actin
