# Supplementary material for: Spatial and temporal dynamics of cancer-associated fibroblast niches in breast cancer
Source: Breast Cancer Res. 2026 Jan 11;28:21. doi: 10.1186/s13058-025-02183-7 (PMC12849564; doi:10.1186/s13058-025-02183-7)
Supplement: Supplementary file 1 — Supplementary Material 1. [file 13058_2025_2183_MOESM1_ESM.docx]

## Addtitional File 1

## 1. Identified CAF substates recapitulate previously described ones.


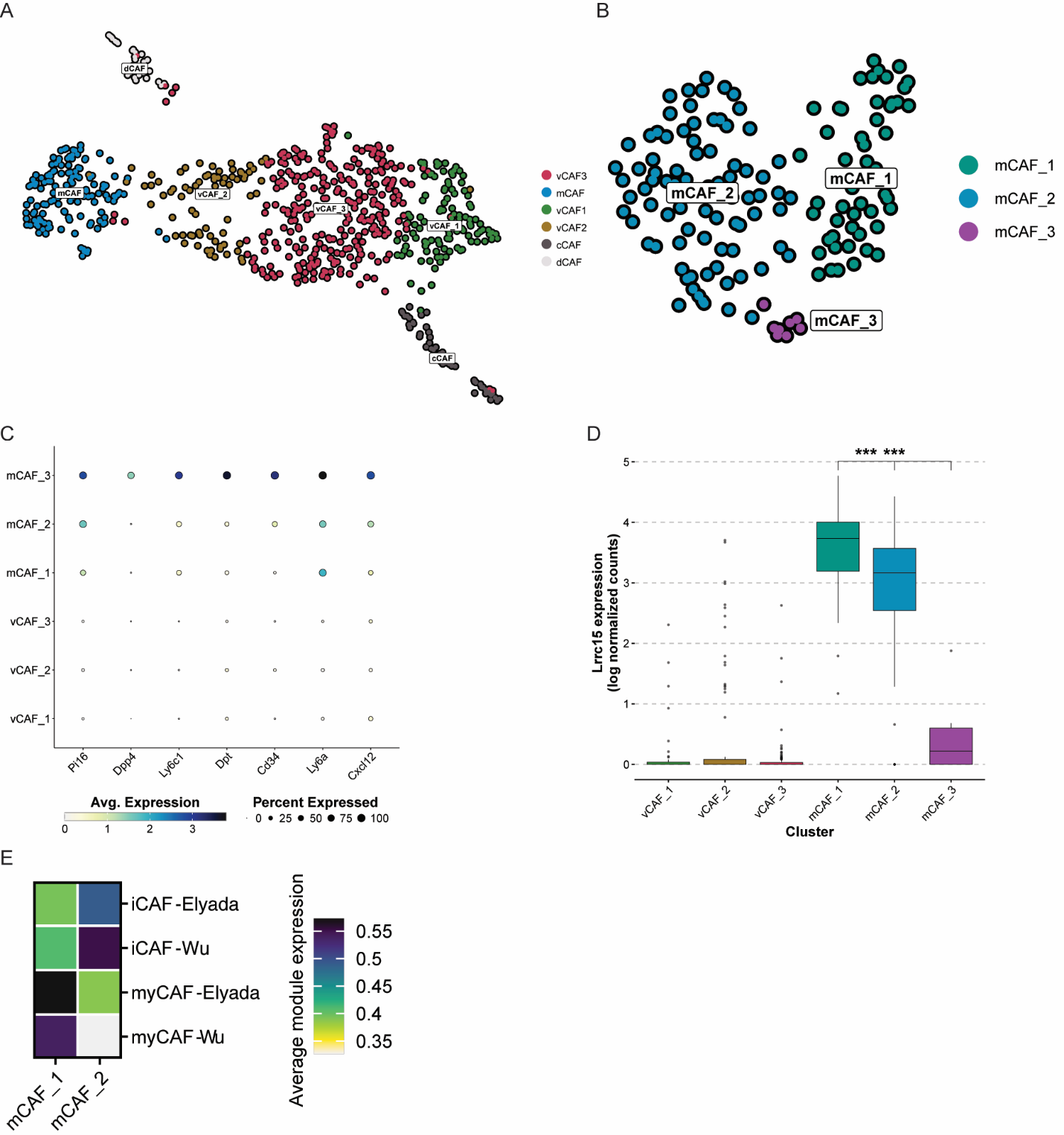


1. UMAP visualization of CAF reclustering.
2. UMAP visualization of mCAFs reclustering.
3. Gene expression dot plot of prototypical progenitor-like normal fibroblast markers.
4. Boxplot of Lrrc15 gene expression levels. Wilcoxon test, **p ≤ 0.01, ***p ≤ 0.001.
5. Heatmap showing the expression of iCAF and myCAF signatures (10, 13) in mCAF1 and mCAF2.
