# Supplementary material for: Spatial and temporal dynamics of cancer-associated fibroblast niches in breast cancer
Source: Breast Cancer Res. 2026 Jan 11;28:21. doi: 10.1186/s13058-025-02183-7 (PMC12849564; doi:10.1186/s13058-025-02183-7)
Supplement: Supplementary file 2 — Supplementary Material 2. [file 13058_2025_2183_MOESM2_ESM.docx]

## 2. mIHC confirms the presence of distinct CAF substates.


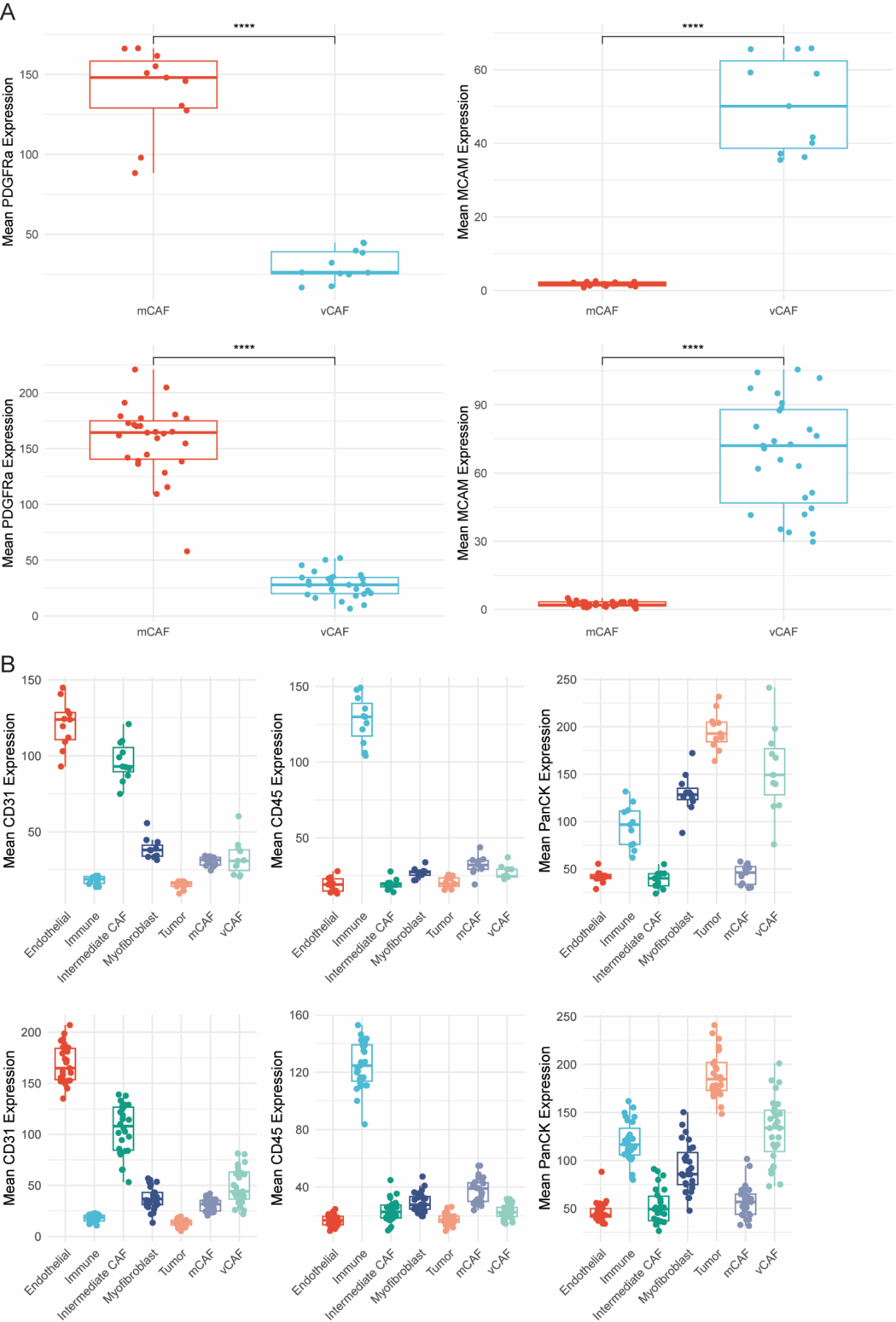


1. PDGFRα and MCAM intensity values in early- (top) and late-stage (bottom) tumors.

B) CD31, CD45, and PanCK intensity values in early- (top) and late-stage (bottom) tumors. Wilcoxon test, ****p ≤ 0.0001. N = 11 in early-stage and n = 27 in late-stage tumors.
