# Supplementary material for: Spatial and temporal dynamics of cancer-associated fibroblast niches in breast cancer
Source: Breast Cancer Res. 2026 Jan 11;28:21. doi: 10.1186/s13058-025-02183-7 (PMC12849564; doi:10.1186/s13058-025-02183-7)
Supplement: Supplementary file 3 — Supplementary Material 3. [file 13058_2025_2183_MOESM3_ESM.docx]

## 3. Image analysis identifies distinct CAF locations and changes in stromal composition across tumor progression.


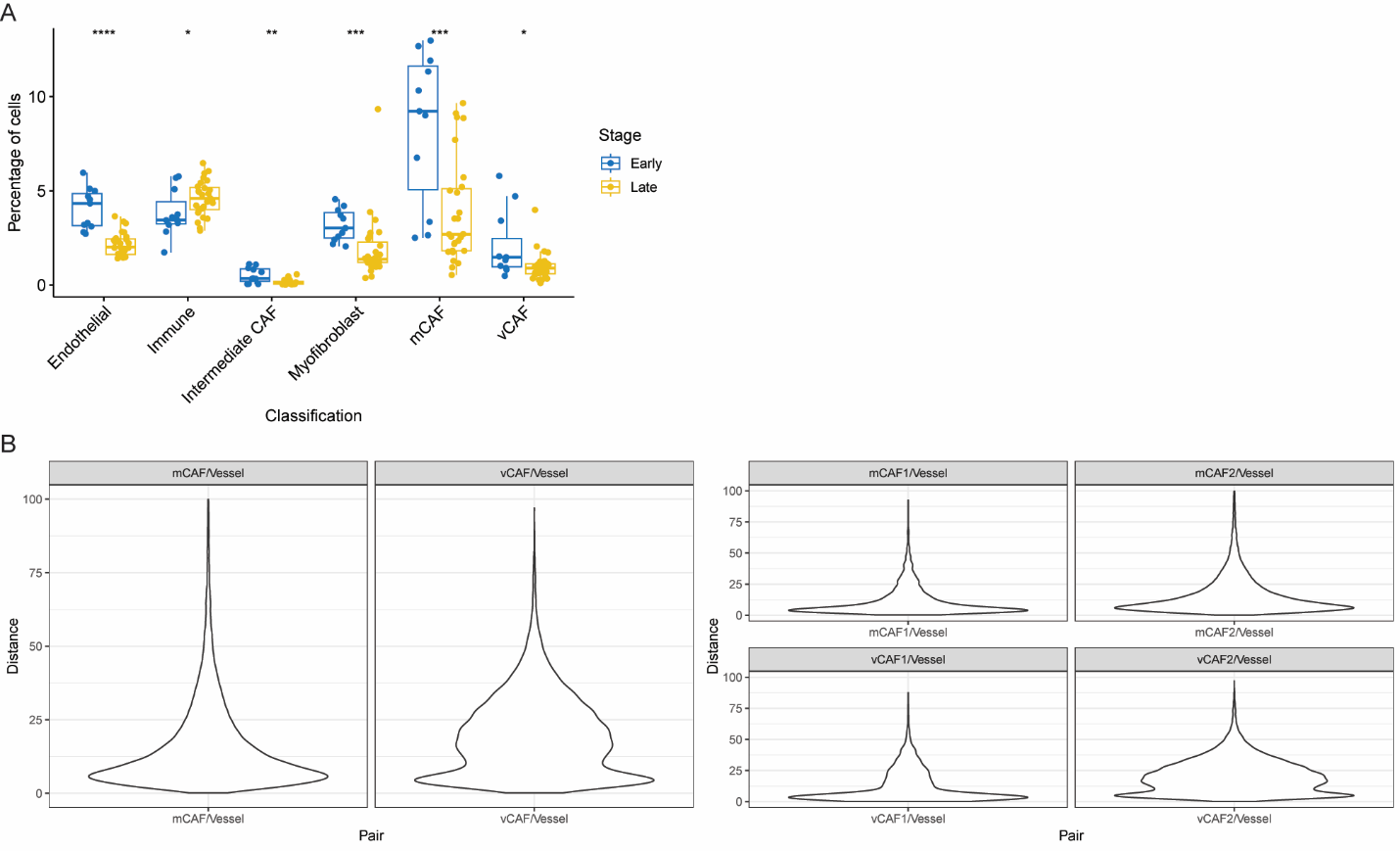


1. Percentages of stromal cells in early and late-stage tumors. N = 11 in early-stage, and n = 27 in late-stage tumors. Wilcoxon test, *p ≤ 0.05, **p ≤ 0.01, ***p ≤ 0.001, ****p ≤ 0.0001.

B) Distribution of distances (µm) from CAFs to the closest endothelial tile centroid in early-stage tumors.
