# Supplementary material for: Spatial and temporal dynamics of cancer-associated fibroblast niches in breast cancer
Source: Breast Cancer Res. 2026 Jan 11;28:21. doi: 10.1186/s13058-025-02183-7 (PMC12849564; doi:10.1186/s13058-025-02183-7)
Supplement: Supplementary file 4 — Supplementary Material 4. [file 13058_2025_2183_MOESM4_ESM.docx]

## 4. CytoMAP neighborhoods clustering identifies distinct regions.


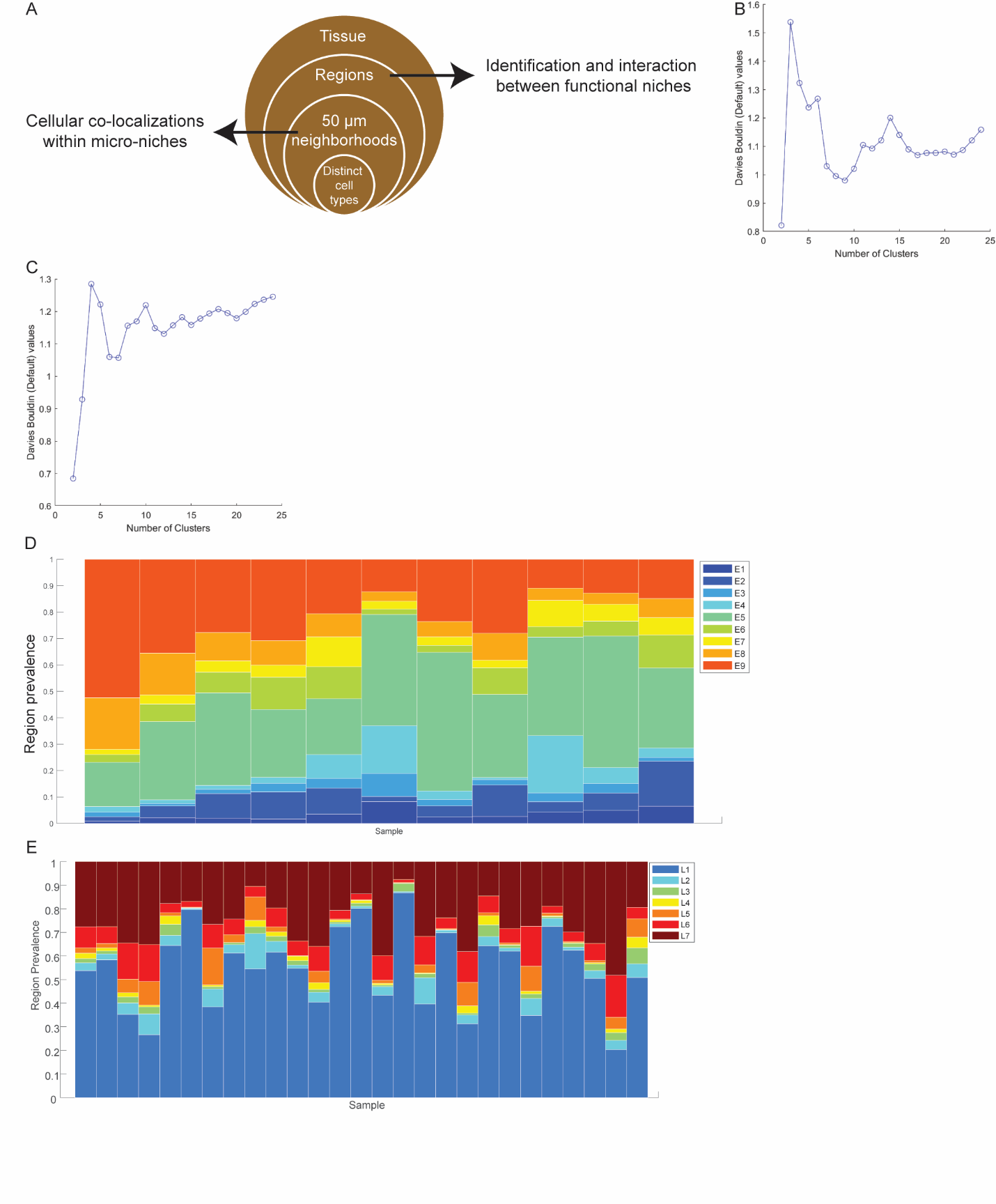


1. Schematic representation of the hierarchical spatial analysis workflow.

B-C). Davies Bouldin values for SOM neighborhoods clustering of early- (B) and late-stage (C) tumors.

D-E) Stacked bar plot of cluster prevalences in each early- (D) and late-stage tumors (E).

N = 11 in early-stage, and n = 27 in late-stage tumors.
