# Supplementary material for: Spatial and temporal dynamics of cancer-associated fibroblast niches in breast cancer
Source: Breast Cancer Res. 2026 Jan 11;28:21. doi: 10.1186/s13058-025-02183-7 (PMC12849564; doi:10.1186/s13058-025-02183-7)
Supplement: Supplementary file 5 — Supplementary Material 5. [file 13058_2025_2183_MOESM5_ESM.docx]

## 5. CytoMAP neighborhood clusters have different cellular composition and interactions.


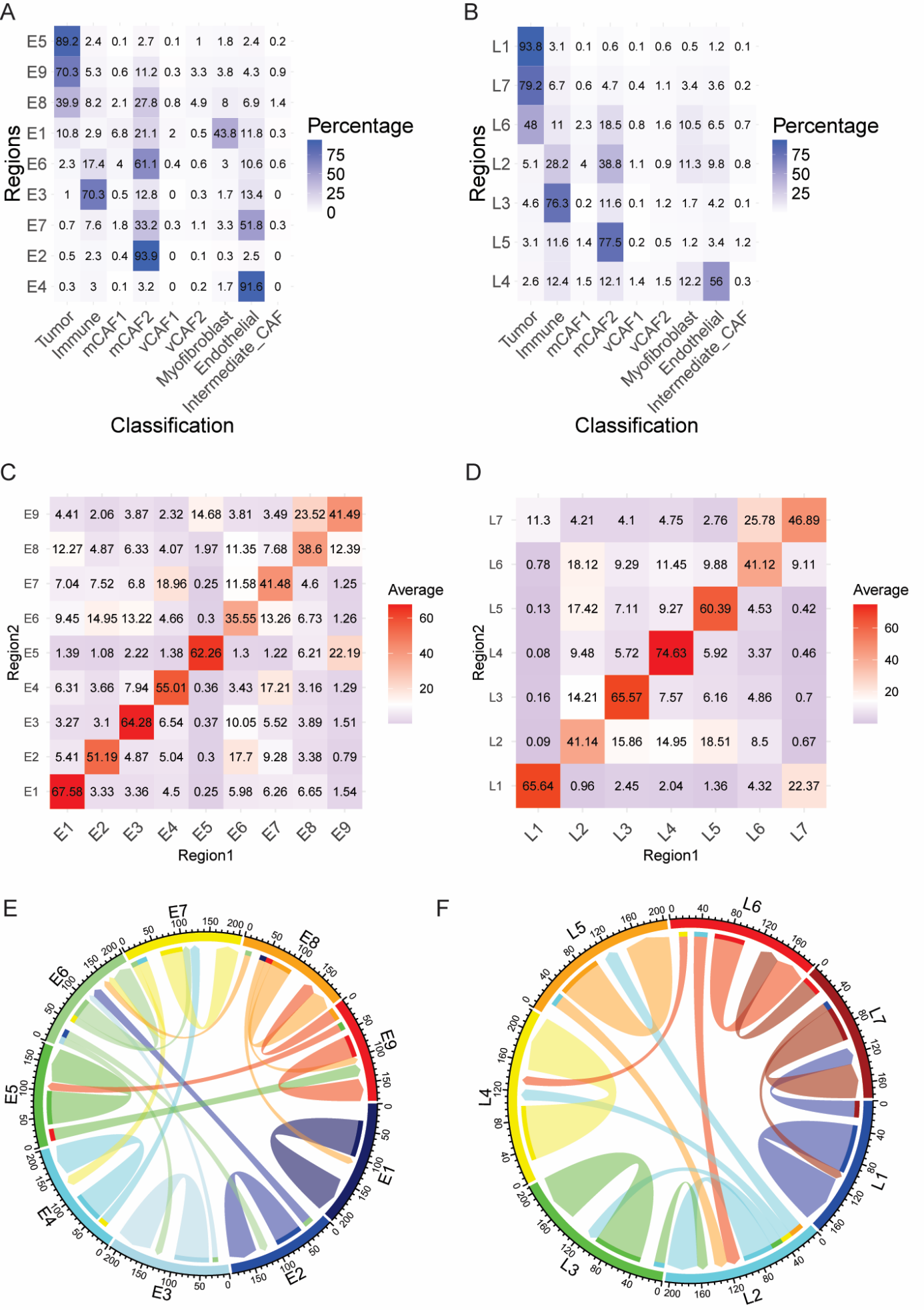


A-B) Heatmap of cluster cellular compositions in early- (A) and late-stage (B) tumors.

C-D) Heatmap of percentages of shared borders between early- (C) and late-stage (D) neighborhood clusters.

E-F) Chord diagram representing percentages of shared borders between regions in early- (E) and late-stage (F) tumors.

N = 11 in early-stage, and n = 27 in late-stage tumors.
