# Supplementary material for: Spatial and temporal dynamics of cancer-associated fibroblast niches in breast cancer
Source: Breast Cancer Res. 2026 Jan 11;28:21. doi: 10.1186/s13058-025-02183-7 (PMC12849564; doi:10.1186/s13058-025-02183-7)
Supplement: Supplementary file 6 — Supplementary Material 6. [file 13058_2025_2183_MOESM6_ESM.docx]

## 6. Validation in human Xenium panel recapitulates the spatial organization observed in murine tumors.


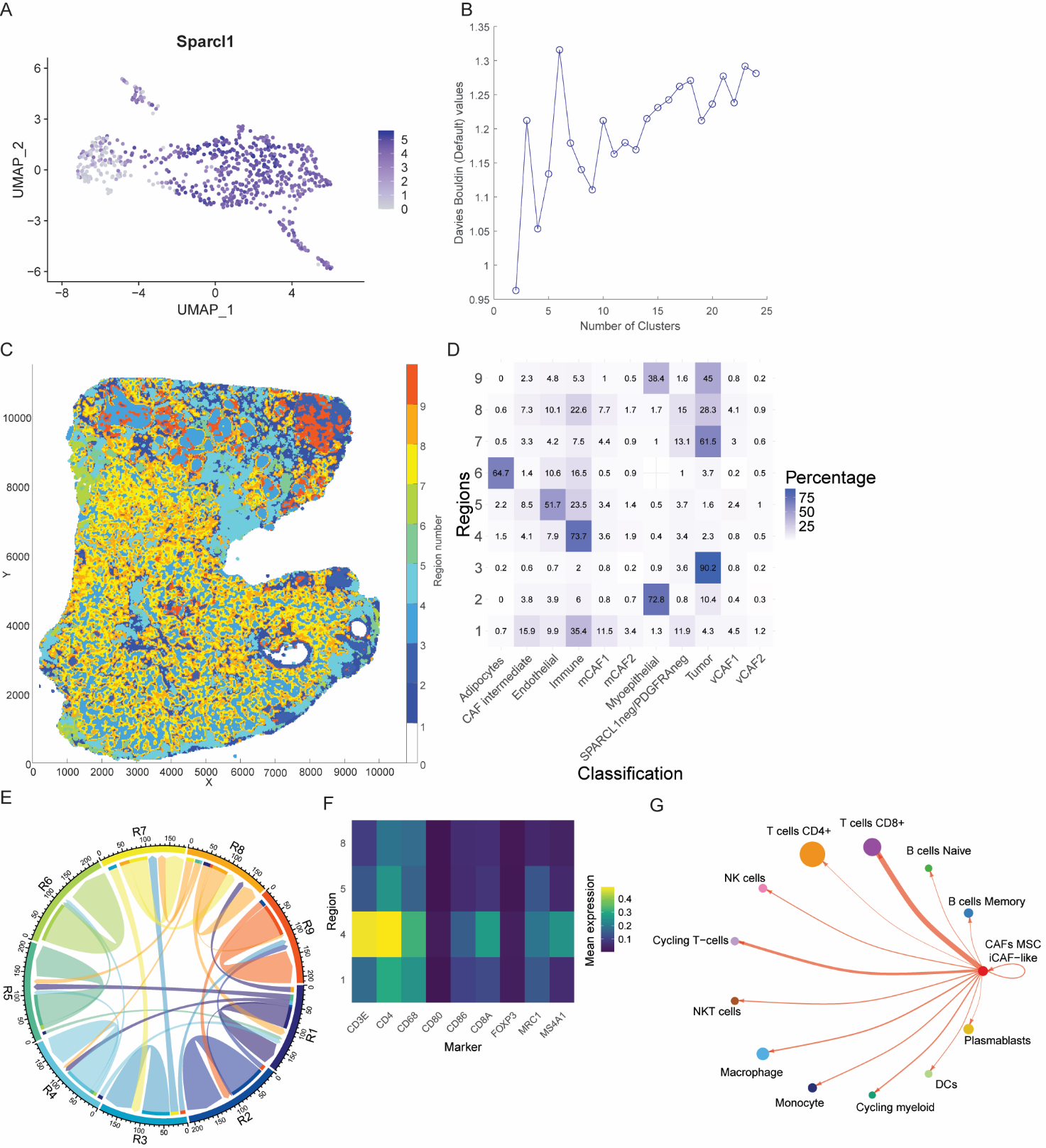


1. Expression of Sparcl1 in our previously published scRNA-seq dataset [1].
2. Davies Bouldin values for SOM neighborhoods clustering.
3. Region color-coded positional plots of the identified neighborhoods.
4. Heatmap of cluster cellular compositions.
5. Chord diagram representing percentages of shared borders between regions.
6. Gene expression values of immune markers in regions enriched in vCAF2 and mCAF2.

G) CellChat circle plot representing interaction strengths of outgoing signals from CAFs MSC iCAF-like from Wu et al. [18]
